# Effects of Neonatal Systemic Inflammation on Blood-Brain Barrier Permeability and Behaviour in Juvenile and Adult Rats

**DOI:** 10.1155/2011/469046

**Published:** 2011-03-10

**Authors:** H. B. Stolp, P. A. Johansson, M. D. Habgood, K. M. Dziegielewska, N. R. Saunders, C. J. Ek

**Affiliations:** ^1^Department of Pharmacology, University of Melbourne, Medical Building 181, Parkville VIC 3010, Australia; ^2^Department of Physiology, Anatomy and Genetics, University of Oxford, Oxford OX13QX, UK; ^3^Institute of Stem Cell Research, Helmholtz Zentrum München, 85764 Neuherberg, Germany

## Abstract

Several neurological disorders have been linked to inflammatory insults suffered during development. We investigated the effects of neonatal systemic inflammation, induced by LPS injections, on blood-brain barrier permeability, endothelial tight junctions and behaviour of juvenile (P20) and adult rats. LPS-treatment resulted in altered cellular localisation of claudin-5 and changes in ultrastructural morphology of a few cerebral blood vessels. Barrier permeability to sucrose was significantly increased in LPS treated animals when adult but not at P20 or earlier. Behavioural tests showed that LPS treated animals at P20 exhibited altered behaviour using prepulse inhibition (PPI) analysis, whereas adults demonstrated altered behaviour in the dark/light test. These data indicate that an inflammatory insult during brain development can change blood-brain barrier permeability and behaviour in later life. It also suggests that the impact of inflammation can occur in several phases (short- and long-term) and that each phase might lead to different behavioural modifications.

## 1. Introduction

Human data related to disorders such as autism, schizophrenia, and cerebral palsy indicate that a period of infection/inflammation during specific stages of brain development may act as a triggering insult [1–4]. In animal experimental studies, inflammation induced during the early postnatal period in rodents has been associated with increased blood-brain barrier permeability [5], white matter damage [6–13], ventricular enlargement [9, 14], and reduced neuron numbers in regions of the hippocampus and cerebellum [15, 16]. In addition, in animals exposed to inflammation *in utero* or during early postnatal life, long-term behavioural alterations such as deficits in prepulse inhibition test [17, 18], motor behaviour [19], and learning and memory [19, 20] have also been reported. However, the biological mechanisms involved in these pathologies are still not understood. To date there are no studies that directly investigated possible links between changes in blood-brain barrier permeability and behavioural alterations in animals exposed to an inflammatory mediator during early stages of brain development. 

In this study, we have investigated possible correlations between some behavioural tests and blood-brain barrier morphology and permeability in adolescent and adult rats that were exposed to a prolonged inflammatory stimulus (LPS-injections) as neonates. To examine possible cellular mechanisms behind the alteration in blood-brain barrier permeability, the distribution of claudin-5, a key tight junction protein shown to directly affect barrier permeability [21, 22], was visualised by immunocytochemistry and electron microscopy was used to study the ultrastructure of brain blood vessels in these animals.

## 2. Materials and Methods

### 2.1. Animal Model

All experiments were approved by the University of Melbourne Animal Ethics Committee according to NH&MRC guidelines. Sprague-Dawley rats were sourced from the Breeding Research Facility at University of Melbourne and all animals were kept under similar condition in standard animal cages with sawdust bedding, free access to food (Specialty feed rat pellets)/water, and under controlled environment (12 h day/light cycle, 20-21°C and 50–60% relative humidity). During the light period, the illumination in the room was >350 lux (1 meter above ground) and background noise due to air-conditioning was about 55 dB.

Newborn rats were given five 0.2 mg/kg intraperitoneal (i.p.) injections of lipopolysaccharide (LPS, *E. coli* 055:B5) or equal volume of sterile saline (control animals) at postnatal day 0 (P0), 2, 4, 6, and 8, to produce a prolonged period of inflammation over the postnatal period [11]. Litters were marked and equally divided into saline- and LPS-treated cohorts, including both male and female animals. During the period of treatment (P0–P8), LPS-injected animals had a significantly lower body weight compared to saline-injected controls. At P8 LPS-treated pups weighed 18.1 ± 0.6 g compared to 22.8 ± 0.7 g for controls (mean ± SEM; *n* = 29 for each group). The body weight was still lower at P20 in LPS-injected animals (controls 65 ± 2 g, 56 ± 2 g for LPS-treated animals; ***P = .0003, *n* = 20 for each group), whereas no significant difference was found in adults (controls 224 ± 14 g, LPS-treated animals 239 ± 16 g; *P* = .51, *n* = 11 for each group).

Animals were left until either P20 or adulthood for behavioural testing; blood-brain barrier permeability measurements were made at P9 and P20 (we have previously published adult sucrose permeability data [11]), claudin-5 immunocytochemistry was performed at P9, P20 and adult; ultrastructural examination of cerebral blood vessels was carried out on adult material only.

### 2.2. Behavioural Tests

All behavioural tests were performed at the Integrative Neuroscience Facility, Howard Florey Institute. Animals were acclimatised to the facility for a week before testing and 1-2 day recovery periods were allowed between each set of tests. The first two tests were conducted at P20 (*n* = 35, 2 days following weaning) and in adult animals (*n* = 16), whereas the last two tests (Open field, Morris water maze) were carried out in adults only, as P20 animals were too young to cooperate. Different cohorts of animals were tested at P20 and as adults to avoid influences in adult responses due to previous exposure to behavioural testing conditions. Rats were tested in random order and under similar environmental conditions to those under which they were normally kept unless specifically stated below.

#### 2.2.1. Prepulse Inhibition and Acoustic Startle Response

For this test the animal was placed in a 9 cm diameter cylinder on a movement-sensitive platform inside a sound-attenuating box with a background sound level of 70 dB. Over 40 minutes a programme of randomly ordered sound stimuli, with 25 second intervals, was given. These included the 115 dB, 40 msec startle stimuli either by itself or preceded by a 100 msec weak prepulse nonstartling stimulus at 74, 78, or 86 dB (i.e., 4, 8 and 16 dB above background), and no stimuli periods. The same programme of sound stimuli was used for each animal. The startle response (a jumping reflex that lasts for less than one second) was measured by STARTLE software.

#### 2.2.2. Light/Dark Test

Animals were placed in a 40 × 40 cm arena, divided into a light (750 lux) and dark (no light) half by a Perspex insert, with a Tru scan locomotor system (light sensors measuring movement in the vertical and horizontal planes) and Tru scan software for live data recording and analysis. After 10 minutes the session was stopped and the animal was returned to its cage. The movement of the animal was analysed including the latency of the first entry and number of entries into the light half.

#### 2.2.3. Open Field Exploration Test

The general locomotor activity of the adult animals was determined in open field exploration. Animals were placed in a 40 × 40 cm arena setup with a Tru scan locomotor system and Tru scan software for live data recording and analysis. After 1 hour the session was stopped and the animal returned to its cage. Analysis of movement, detected by the Tru Scan locomotor system, was conducted for horizontal and vertical planes as well as the movement of the animal along the margins or the centre of the arena.

#### 2.2.4. Morris Water Maze

A circular water maze pool (2 m diameter) was filled to a depth of 30 cm with 25°C water and enough nontoxic paint to make the water opaque. A circular 15 cm diameter platform was submerged 1 cm below the water level in one quadrant of the pool. The animal was placed in the water in a random quadrant and allowed 2 minutes to find the platform. The time to find the platform was recorded. The animal was left for 30 seconds on the platform before being removed, dried and placed under a warming lamp before repeating the test with the same platform location, but the animal entering the pool in a different quadrant. The testing procedure was repeated daily until the animals showed no improvement in the time taken to find the platform. For a final test, the platform was removed and the animal was introduced to the pool for 1 minute. The frequency and duration the animal spent in the platform zone (the zone where the platform used to be) were measured.

### 2.3. Blood-Brain Barrier Permeability

Brain/plasma sucrose concentration ratios were measured as an index of blood-brain barrier permeability in animals that had been injected with either LPS or saline as neonates (see above). Due to the small size of P9 animals, different methods were used to estimate concentration ratios at younger (P9) and older (P20) ages (*n* = 6 for each age group and treatment).

In P9 animals, serial blood sampling is not possible therefore, in order to obtain proper steady state concentration ratios, nephrectomy was performed under isoflurane (3%) anaesthesia before an i.p. injection (6 *μ*L/g body weight) of 1 *μ*Ci ^14^C-sucrose (Amersham, CFB146). Animals regained consciousness and were kept under a heat-lamp at 28°C. Three hours after the sucrose injection, when concentration ratios achieve a near to steady state level [23], the animals were terminally anaesthetised with halothane and blood and brains collected. Plasma was separated by centrifugation. The brain was divided into cerebral hemispheres, midbrain, cerebellum and brainstem and frozen before further processing. 

In P20 rats serial blood sampling was possible, and was used to construct the plasma radioactivity curve following radiolabel injection [11]. In anaesthetised animals, the left femoral vein and artery were cannulated, and 2 *μ*Ci (P20) of ^14^C-sucrose was injected (6 *μ*L/g body weight) via the venous cannula, followed by an equal volume of saline. Blood was collected (60–80 *μ*L) from the arterial cannula into heparinised glass capillaries every 5 minutes until the end of the experiment (30 minutes) and plasma separated by centrifugation. Immediately after the last blood sample was collected the heart was transected to prevent further circulation of the tracer and the brain dissected out. The brain was divided into the cerebral hemispheres, midbrain, cerebellum and brainstem and frozen.

Soluene-350 (0.5 mL, Packard Biosciences) was added to each vial with brain tissue and left for 48 hours at 37°C to completely solubilise the tissue. Following this, 4.5 mL of scintillation fluid (Ultra Gold, Packard Biosciences) was added to all vials with brain or plasma samples. The radioactivity in each vial was determined by liquid scintillation counting (1409 DSA, Wallac) and expressed as dpm/*μ*g sample. Brain/plasma sucrose concentration ratios were calculated as has been described before [11]. We have previously measured permeability in adult animals using the same treatment protocol and methods as for P20 animals [11], and data are presented along with the results from this study.

### 2.4. Claudin-5 Immunocytochemistry

LPS or saline-injected animals at P9, P20 and adult were terminally anaesthetised with inhaled isoflurane (P9) or i.p. Nembutal (P20 and adults; 0.1-0.2 mL/100 g, Rhone Merieux) before perfusion through the aorta with heparinised phosphate buffered saline (PBS, pH 7.4) followed by 4% paraformaldehyde in 0.1 M phosphate buffer (pH 7.4). Brains were removed from the skull, postfixed in Bouin's fixative for 24 hours and processed and sectioned for histology. Briefly, tissue was dehydrated through increasing concentrations of ethanol, cleared in chloroform and embedded in paraffin wax. Sections were cut coronally with a thickness of 5 *μ*m on a Bright rotary microtome (Leica) [5, 11]. 

Claudin-5 protein was detected in paraffin embedded tissue sections using the PAP method of immunohistochemistry. Sections were dewaxed, rehydrated and incubated in Peroxidase Blocker followed by Protein Blocker (1 hour each; Blockers sourced from DAKO). Sections were exposed to the primary antibody, mouse anticlaudin-5 (Zymed, diluted 1 : 200) overnight at 4°C. Secondary rabbit antimouse antibody and mouse-PAP (DAKO) were used following standard procedures [11] and sections developed for approximately 5 minutes using the DAB+ kit (DAKO). Between each incubation step, sections were washed 3 × 5 minutes with PBS-Tween20 (pH 7.4). Controls included incubating slides with one of the antibodies omitted and were always blank.

 Immunocytochemical distribution of claudin-5 in microvessels was determined under light microscope to be either “junctional” or “cytoplasmic” as described previously [11]. The proportion of vessels with either appearance in the cortex and white matter (corpus callosum and external capsule) was determined and scored by the same blinded observer (KMD, 3–6 sections were counted per brain, *n* = 3 brains in each group).

### 2.5. Electron Microscopy

For morphological examination of blood vessels with an electron microscope tissue was collected from adult rats exposed to either LPS or saline as neonates (*n* = 3-4 for each group). Animals were anaesthetised as described above and perfused transcardially with 50 mL heparinised phosphate buffered saline followed by 200 mL solution of 2% paraformaldehyde/2.5% glutaraldehyde in phosphate buffer (0.1 M, pH 7.3). Several tissue blocks (up to 1 mm^3^a piece) were cut from different parts of the corpus callosum and external capsule from each animal and postfixed overnight in the same fixative. Tissue was washed in sodium cacodylate buffer (0.1 M, pH 7.3, 3 × 20 minutes) before processing with a 2% osmium tetroxide/1% potassium ferricyanide solution followed by uranyl acetate treatment. Tissue was dehydrated in increasing concentrations of acetone and embedded in Araldite Epon. Ultrathin sections were cut with a Reichard UltraE microtome and contrasted with uranyl acetate and lead citrate. Thirty to fifty blood vessels from each tissue block were examined under a Phillips CM10 electron microscope to determine their ultrastructure with special focus on the integrity of tight junctions.

### 2.6. Statistical Analysis

Comparisons between saline-(control) and LPS-(experimental) injected animals were made using regression analysis for the prepulse inhibition test. For all other data, differences between control and experimental animal groups were compared for statistical significance using Student's *t*-tests with corrections for multiple comparisons. A *P*-value of less than .05 was considered significant in all cases.

## 3. Results

### 3.1. Behavioural Tests

The effect of systemic inflammation during early postnatal development of the rat on behaviour in later life was determined using behavioural tests previously shown to be associated with early life exposure to inflammation, such as anxiety (light/dark test) and altered prepulse inhibition (PPI) as well as general tests of motor behaviour and learning. 

#### 3.1.1. Prepulse Inhibition and Acoustic Startle Response

**Figure 1:**
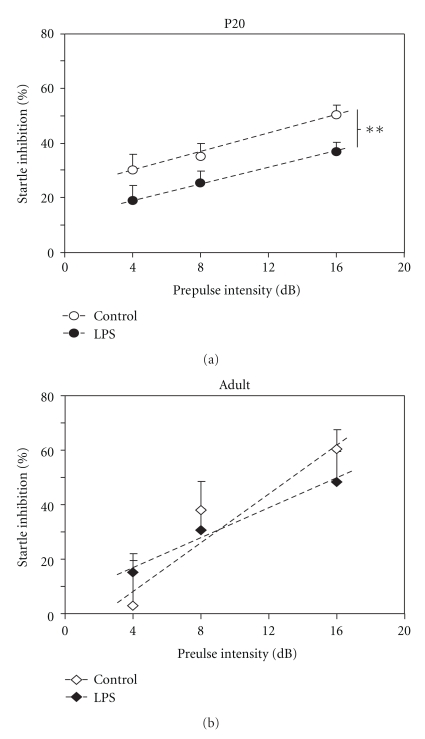
The sensory motor gaiting of animal cohorts was tested using the prepulse inhibition paradigm. Animals were exposed to a 115 dB stimulus either alone or with a 4, 8 or 16 dB prepulse. The presence of a prepulse inhibits the startle response produced by the 115 dB stimulus alone. Data are presented as percent startle inhibition (mean ± SEM) for each prepulse intensity. At P20 (a), animals exposed to LPS had reduced startle inhibition compared to saline-injected age-matched controls (**P < .01, linear regression analyses; *n* = 29). Adult animals (b) showed no significant difference between groups of animals although there was a trend toward reduced startle inhibition at 16 dB prepulse in LPS-treated animals (*n* = 8 for each data point).

Acoustic startle response and prepulse inhibition were measured as part of one test. No significant difference in acoustic startle response was observed between LPS-treated or control animals at either P20 or adult (data not shown). However, a significant decrease in prepulse startle inhibition was observed in LPS-treated animals at P20 (linear regression analysis, **P < .01, Figure 1(a)). In adult animals there was a trend towards a decreased prepulse inhibition at the higher prepulse intensity (16 dB), the difference was not statistically significant (Figure 1(b)).

#### 3.1.2. Light/Dark Test

**Figure 2:**
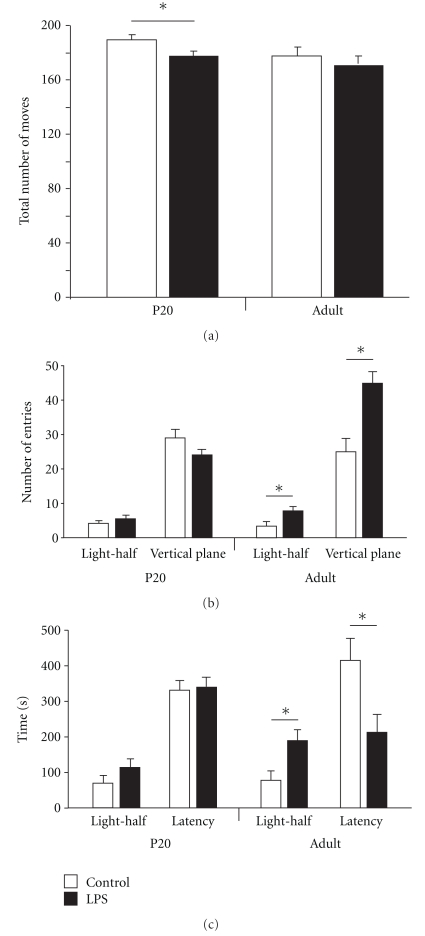
The behaviour of P20 and adult animals in a stressful environment was determined in an arena where half was brightly lit and the other half was dark and enclosed. Total moves (a) and entries into the light half and the vertical plane were determined (b), as well as time spent in the light half and latency (c). P20 animals exposed to LPS during postnatal development showed a small but significant decrease in total movement in this apparatus, but otherwise showed no changes compared to saline-injected controls (*n* = 8 for each group). Adult animals treated with LPS showed increased exploratory behaviour with increased time and entries into the light half of the arena, reduced latency to enter the light half and increased vertical plane entries (*P < .05, data are mean ± SEM, *n* = 8 for each group).

P20 LPS-treated animals showed a small but significant decrease in their total moves in the Light/Dark test (*P = .02, Figure 2(a)) compared to those treated with saline. However, no changes in their entrance into the light half of the apparatus, the time spent in the light half or the latency to enter the light half was detected (Figures 2(b) and 2(c)). In contrast, in adult animals LPS treatment resulted in significantly increased entries into the light half of the apparatus as well as earlier entry and more time spent in the light half of the chamber (Figures 2(b) and 2(c)). They also showed generally more exploratory behaviour, as indicated by increased vertical plane entries (Figure 2(b)).

#### 3.1.3. Open Field Exploration Test

LPS-treated adult rats showed no difference from control animals in any of the parameters analysed such as the total number of moves (884 ± 62 versus 895 ± 60), time (sec) moving (1321 ± 127 versus 1383 ± 136), or in their velocity (cm/sec, 1522 ± 209 versus 1508 ± 223).

#### 3.1.4. Morris Water Maze

**Figure 3:**
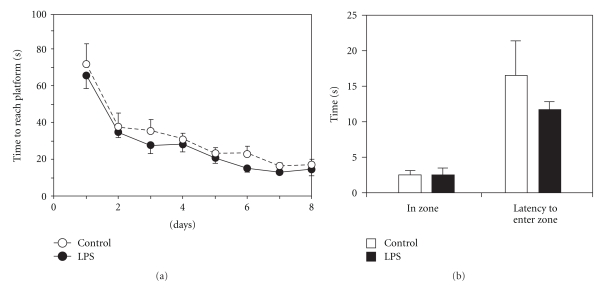
Adult animals treated with either saline or LPS during the postnatal period were tested for their ability to learn and remember and their general locomotor activity in the Morris Water Maze. No difference was observed between saline- and LPS-treated animals for any of the measured parameters. Data are mean ± SEM, *n* = 8 for each group.

Adult animals exposed to LPS during their early postnatal development showed no significant differences in behaviour from the saline-injected controls in the Morris Water Maze, with no changes in the time taken to learn the location of a submerged platform or their exploratory behaviour when the platform was removed (Figures 3(a) and 3(b)).

### 3.2. Blood-Brain Barrier Permeability

Results from permeability studies performed in this paper are only valid within each age group and cannot be directly compared between P9 and older animals since different methods were used at different ages to determine brain/plasma sucrose concentration ratios (see Methods). Results are presented in Table 1. At P9 and P20 there was no significant difference in permeability of the blood-brain barrier between the LPS and saline-treated groups of animals. At P9 the brain/plasma sucrose concentration ratios of control animals were 12.5 ± 0.6% (mean ± SEM) compared to 11.9 ± 1.6% in LPS-treated animals (*P* = .8). At P20 the brain/plasma sucrose concentration ratios in controls were 2.6 ± 0.2% compared to 2.1 ± 0.1% (*P* = .1) in LPS-treated animals (see Table 1). In contrast, in adult animals we have shown previously [11] there is a significant increase in the brain/plasma sucrose concentration ratios following early postnatal LPS treatment (4.2 ± 0.5% in LPS-treated animals compared to 2.5 ± 0.4% for controls, *P < .05).

### 3.3. Claudin-5 Immunocytochemistry

**Figure 4:**
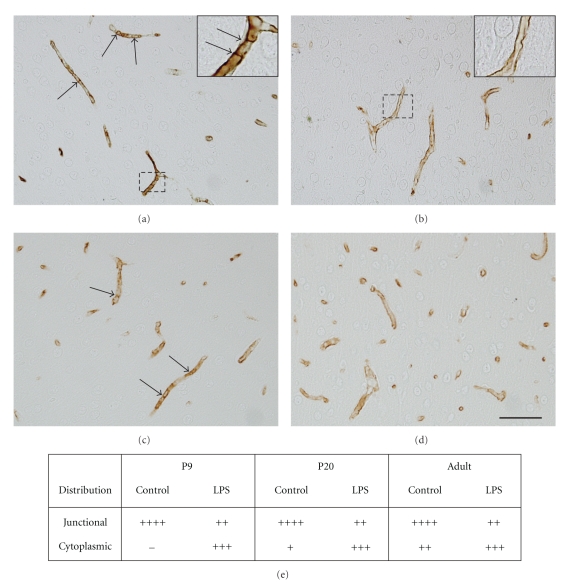
Claudin-5 immunoreactivity was detected on the endothelial cells of brain microvessels. In P9 (a) and adult (c) control brains, claudin-5 (brown reaction product) appears to be continuous along the length of the microvessels and to form a distinct pattern consistent with its location at cell-to-cell junctions (higher magnification in insert in (a)). In P9 (b) and adult (d) animals treated with LPS during the first 8 days of life a proportion of vessels in the brain (see also (e)) showed an altered distribution of claudin-5, with a fainter immunoreactivity and more diffuse staining pattern apparently in the cytoplasm (see insert for cellular distribution pattern in insert in (b)). Scale bar = 50 *μ*m. Inserts show magnification of regions indicated by box in (a) and (b). (e) illustrates a relative proportion of cerebral blood vessels displaying claudin-5 immunoreactivity that was observed as “junctional” or cytoplasmic (see Methods). This showed that there is a shift towards cytoplasmic staining of claudin-5 in LPS-treated animals at all ages. *n* = 3 for each group.

Claudin-5 is so far the only tight junction protein that has been experimentally shown to directly affect the permeability of the blood-brain barrier [22], therefore its distribution was investigated using immunocytochemistry on paraffin-embedded brain sections (see Methods) and the results are illustrated and summarised in Figure 4. In saline-injected control animals at all ages studied (P9, P20 and adult), claudin-5 immunohistochemistry showed a typical cell-to-cell junction distribution pattern (Figures 4(a) and 4(c)) in approximately 80–100% of vessels (indicated as ++++ in Figure 4(e)) within the cortex and white matter while in the remaining cerebral blood vessels distribution of the claudin-5 immunoreactivity appeared more cytoplasmic. Distribution of claudin-5 immunoreactivity in brains from all LPS-treated animals showed a distinct shift, typically exhibiting less junctional staining and a relatively more cytoplasmic distribution (see Figures 4(b), 4(d), and 4(e)). 

### 3.4. Ultrastructure of Cerebral Blood Vessels

**Figure 5:**
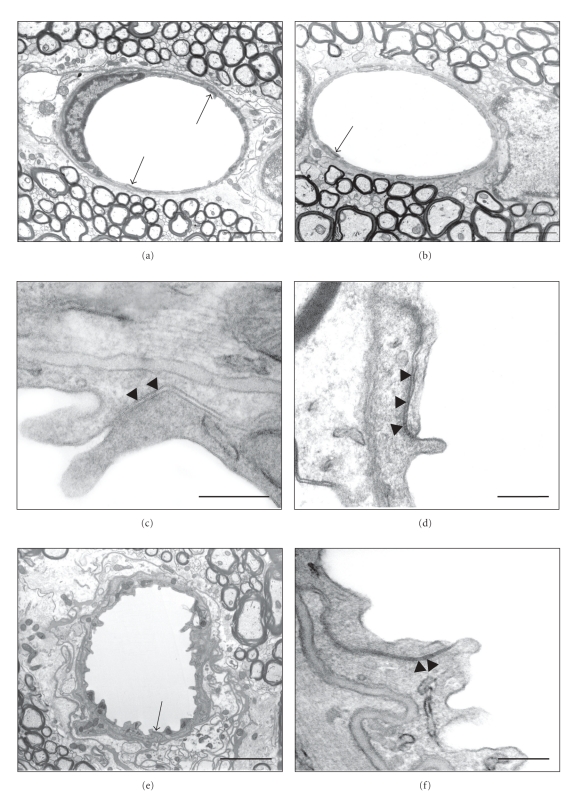
Electronmicrographs of blood vessels in the external capsule of adult control and LPS-treated rats. Most blood vessels appear identical in control (a) and LPS-treated animals (b), arrows indicate tight junctions. At higher magnification the tight junctions are visible and these look normal in LPS-treated animals (d) compared to control animals (c) with several fusions points (arrowheads) along the intercellular cleft. A small proportion of cerebral capillaries in LPS-treated animals appeared abnormal with convoluted lumen and poorly structured perivascular space (e). In these vessels the tight junction (arrow in (e)) still appeared normal and at higher magnification the characteristic fusion points were visible within the tight junction (arrowheads in (f)). Scale bars: 3 *μ*m in (a), (b), (e); 200 nm in (c), (d), (f).

The ultrastructure of blood vessels, and specifically tight junctions, in the white matter (external capsule and corpus callosum) was examined. Tissue was compared from adult control animals and adult animals that had been exposed to LPS during development. All vessels examined from control brains appeared to have a normal ultrastructure, with little perivascular space, close association of astrocytic end feet, well defined basement membrane (Figure 5(a)) and obvious tight junctions between the intercellular clefts of apposing endothelial cells (Figure 5(c)). Most vessels in white matter from LPS-treated animals were not obviously different from control brains (Figure 5(b)). Tight junctions were apparent at cell-cell contacts (Figure 5(d)), as were the associations with the surrounding structures. However, in 2 out of 50 vessels in sections from the LPS-treated animals, ultrastructural abnormalities were observed (Figure 5(e)). In these vessels the lumen was convoluted and there was an apparent disruption of the perivascular space. Despite this, the structure of the tight junctions associated with these unusual vessels appeared to be normal (Figure 5(f)).

## 4. Discussion

Increasing evidence, both clinical and experimental, indicates that an early inflammatory insult can affect brain development and behaviour later in life. The aim of this study was to determine whether there is a correlation between changes in the permeability properties of the blood-brain barrier induced by a period of neonatal inflammation and later behaviour using the rat as an experimental model.

### 4.1. Blood-Brain Barrier Permeability and Behaviour

In the present model of early life inflammation some alterations in blood-brain barrier function and white matter damage have previously been reported. These include short-term changes in blood-brain barrier permeability to protein in young pups and long-term changes to small molecular weight molecules in adult animals exposed to prolonged inflammation during development [11]. The present study determined that this long-term permeability change develops sometime after 3 weeks of age, as animals tested at P20 showed no significant change in the permeability of the blood-brain barrier to sucrose (Table 1). Interestingly, changes in blood vessel tight junction distribution preceded long-term alterations in both blood-brain barrier permeability and behaviour. 

It has previously been hypothesised that this alteration in function of the blood-brain barrier in the adult may contribute to inflammation-induced behavioural changes such as altered prepulse inhibition (PPI). The results from the current study suggest that changes in PPI occur prior to the long-term change in barrier function to small molecules, however they may relate to damage resulting from the transient increase in protein permeability and white matter volume reduction that occurs up to P9 [11].

One of the major behavioural modifications observed in the present study was a change in sensory-motor gaiting, demonstrated by PPI test in juvenile rats, but not in adults following an early inflammatory insult (Figure 1). Changes in PPI have been reported in several studies that examined effects of systemic inflammation during fetal or early postnatal stages of brain development, however, some results have been conflicting. Fortier and colleagues [17] found that LPS administered to pregnant mice during mid to late gestation resulted in reduced PPI in offspring when adult, whereas polyI:C, which is used to mimic viral infections, did not. In contrast, higher doses of polyI:C at comparable ages of gestation have been found to result in altered PPI in a separate study [24]. Similarly, Fortier et al. [17] suggested that inflammation at very early stages of gestation (GD10-11) in the mouse did not cause changes in the PPI response of the adult offspring, while an earlier study by Shi et al. [18] indicated that inflammation-induced either by viral infection or injection of the polyI:C viral mimic at GD9.5 did in fact result in a reduced PPI response in adult offspring. These discrepancies could be due to the different doses of polyI:C used, particularly as inflammation has been associated with increased serum cortisol concentration in the perinatal period [25], which in turn has been found to alter PPI in a dose-specific manner [26]. This suggests that these two systems may interact to produce widely different results depending on the magnitude of the initial insult. Therefore, while changes in PPI are now frequently seen in models of early life inflammatory challenge, the long-term behavioural outcome is dependent on many factors, including dose of inflammatory agent and gestational age at time of insult. The present study supports the recent finding of Ibi et al. [27] that neonatal inflammation, in comparison to inflammation during gestation, also results in long-term alteration in the PPI response. It also supports the hypothesis of Wolff and Bilkey [24] that PPI deficits are distinguishable in juvenile animals. 

Behavioural changes seen at P20 for PPI may continue into adulthood although in this study the difference between the two treatment groups was no longer statistically significant (Figure 1). LPS challenged animals did however show a significant behavioural change in the Light/Dark test compared to saline-treated animals when adult (Figure 2). In contrast, in animals tested at P20 there was no significant difference for this test between control and LPS-injected animals suggesting that the effect seen in adult animals may take a longer period to develop and is not manifested until later in life. However, it may be that this is not an appropriate test for such juvenile animals, as many of them (both saline- and LPS-injected) did not enter the light area of the arena at all during the testing period.

The results of the Light/Dark test obtained for adult animals were different from what might have been expected from clinical disease associations [28, 29] and some previous studies. Fetal exposure to inflammation (via the dam) in the C57 mouse produced long-term anxiety-like behaviour on the elevated plus maze [30]. However, a study of inflammation in the postnatal rat (P7-28) did not show any increased anxiety-like behaviour on the same apparatus [31, 32]. The reduced light avoidance exhibited by the LPS-treated animals in the present study (Figure 2) does not appear to be due to reduced sensory input. Animals receiving LPS-injections during infancy responded normally to visual and auditory stimuli in the Morris Water Maze and PPI. It therefore seems that much of the apparent conflict in results so far reported in the literature may reflect differences in timing of inflammation during development, the degree of inflammatory insult, the method of inducing it and perhaps the species. 

LPS-treated animals showed no difference in their behaviour compared to saline-injected control animals for either the open field or the Morris Water Maze, indicating that not all aspects of behaviour are affected as a result of inflammatory insult during early postnatal development.

### 4.2. Tight Junctions

We examined tight junctions both morphologically under the electron microscope and molecularly using claudin-5 immunoreactivity. Tight junctions located between endothelial cells of cerebral blood vessels are the structural basis for the blood-brain barriers that normally severely restrict paracellular permeability from blood into brain [33]. The present study of blood vessel ultrastructure in the white matter of adult animals exposed to LPS early in development showed no apparent alteration in the structure of tight junctions with several “kissing points” visible as an indicator of closely apposed membranes.

However, while examining these junctions we found a small number of the blood vessels that did appear to have gross ultrastructural abnormalities (Figure 5). The gross ultrastructural changes observed in these vessels are not dissimilar to those observed in animal models of pericyte deficiency [34] where increased permeability of the blood-brain barrier was observed. Although the proportion of these abnormal vessels was low this does not rule out their potential to influence blood-brain barrier permeability. Due to the normally extremely low permeability of cerebral blood vessels, a few vessels with altered permeability could significantly contribute to the overall properties of the blood-brain barrier. Although the tight junctions in all vessels appeared to be normal (including those in the abnormal vessels, Figure 5), the functionality of these tight junctions can only be confirmed using electron denser tracers [35]. Sucrose permeability was increased throughout the cortex in these animals, indicating that other low molecular weight molecules, such as some drugs or heavy metals, may have prolonged access to the brain, potentially contributing to long-term damage of the brain.

The tight junctions are composed of a number of proteins that are thought to have different roles in relation to paracellular permeability. Disruption of these proteins has been associated with alterations in blood-brain barrier permeability [36–38] and the expression of, for example, claudin-5 can be regulated through protein kinase C by the actions of a number of cytokines [39]. Molecular alterations of the tight junction proteins may lead to increased permeability even without ultrastructurally altering the junctions. In a claudin-5 knockout mouse no overt morphological abnormalities in the blood vessels were found despite a size selective increase in blood-brain barrier permeability [22]; however, as this study did not include EM immunocytochemistry it is not clear what was the cellular route (paracellular or transcellular) responsible for the observed increase in barrier permeability to small molecules that was observed. Although there is a link between altered claudin-5 distribution and an increase in sucrose permeability in adults in the present study (Table 1), the results from younger animals show that LPS treatment can also cause an altered distribution of claudin-5 protein immunoreactivity that is not associated with changes in sucrose permeability. In order to understand these differences we need a better understanding of the molecular functions of tight junction proteins as well as the changes of these proteins that may occur in response to LPS treatment.

In conclusion, the present results demonstrate that a period of prolonged systemic inflammation in the neonatal rat can cause a multitude of modifications that manifest in later life, ranging from alterations in behaviour, changes in blood-brain barrier permeability and in structure of some cerebral blood vessels. The results also show that these changes develop at different times after the initiating inflammatory insult and are not always temporally correlated. The results from the present study are suggestive of a two-phase progression model: a first wave of damage (i.e., acute blood-brain barrier disruption and white matter damage [11]) that causes early changes in blood vessel morphology/composition and early behavioural alterations and a second phase of damage, induced by the adult-onset increase in blood-brain barrier permeability, that produces the later behavioural changes.

## Figures and Tables

**Table 1:** Brain/plasma sucrose concentration ratios.

Age	Control	LPS
P9	12.5 ± 0.6 (*n* = 6)	11.9 ± 1.6 (*n* = 6)
P20	2.6 ± 0.2 (*n* = 6)	2.1 ± 0.1 (*n* = 6)
Adult	2.3 ± 0.4^∧^ (*n* = 7)	4.2 ± 0.5*^∧^ (*n* = 7)

Note: P9 ratios were from nephrectomised awake animals 3 hours after an i.p. injection, whereas ratios in older animals were 30 min ratios in anaesthetised animals after an i.v. injection. Data are mean ± SEM, *P < .05 from control, ^∧^data published previously [11].
